# Trk inhibition reduces cell proliferation and potentiates the effects of chemotherapeutic agents in Ewing sarcoma

**DOI:** 10.18632/oncotarget.8992

**Published:** 2016-04-26

**Authors:** Tiago Elias Heinen, Rafael Pereira dos Santos, Amanda da Rocha, Michel Pinheiro dos Santos, Patrícia Luciana da Costa Lopez, Marco Aurélio Silva Filho, Bárbara Kunzler Souza, Luís Fernando da Rosa Rivero, Ricardo Gehrke Becker, Lauro José Gregianin, Algemir Lunardi Brunetto, André Tesainer Brunetto, Caroline Brunetto de Farias, Rafael Roesler

**Affiliations:** ^1^ Cancer and Neurobiology Laboratory, Experimental Research Center, Clinical Hospital (CPE-HCPA), Federal University of Rio Grande do Sul, Porto Alegre, RS, Brazil; ^2^ Department of Pharmacology, Institute for Basic Health Sciences, Federal University of Rio Grande do Sul, Porto Alegre, RS, Brazil; ^3^ Faculty of Health Sciences, UniRitter Laureate International Universities, Porto Alegre, RS, Brazil; ^4^ Departament of Pathology, Faculty of Medicine, Federal University of Rio Grande do Sul, Porto Alegre, RS, Brazil; ^5^ Department of Orhopaedics and Traumatology, Clinical Hospital, Federal University of Rio Grande do Sul, Porto Alegre, RS, Brazil; ^6^ Department of Pediatrics, Faculty of Medicine, Federal University of Rio Grande do Sul, Porto Alegre, RS, Brazil; ^7^ Children's Cancer Institute (ICI), Porto Alegre, RS, Brazil; ^8^ Pediatric Oncology Service, Clinical Hospital, Federal University of Rio Grande do Sul, Porto Alegre, RS, Brazil

**Keywords:** TrkA, TrkB, neurotrophin, neurotrophin receptor, Ewing sarcoma

## Abstract

Ewing sarcoma (ES) is a highly aggressive pediatric cancer that may arise from neuronal precursors. Neurotrophins stimulate neuronal devlopment and plasticity. Here, we found that neurotrophins nerve growth factor (NGF) and brain-derived neurotrophic factor (BDNF), as well as their receptors (TrkA and TrkB, respectively) are expressed in ES tumors. Treatment with TrkA (GW-441756) or TrkB (Ana-12) selective inhibitors decreased ES cell proliferation, and the effect was increased when the two inhibitors were combined. ES cells treated with a pan-Trk inhibitor, K252a, showed changes in morphology, reduced levels of β-III tubulin, and decreased mRNA expression of NGF, BDNF, TrkA and TrkB. Furthermore, combining K252a with subeffective doses of cytotoxic chemotherapeutic drugs resulted in a decrease in ES cell proliferation and colony formation, even in chemoresistant cells. These results indicate that Trk inhibition may be an emerging approach for the treatment of ES.

## INTRODUCTION

Tumors of the Ewing sarcoma (ES) family are aggressive childhood cancers [1]. ES remains the second most common primary bone malignancy in the pediatric population, with an annual incidence of almost 3 cases per million people in the USA [2]. These tumors are characterized by highly aggressive, small round blue cells of the bone and soft tissue, genetically marked by gene fusions involving, most commonly, the *EWS* gene and a gene of the *ETS* family (primarily *FLI*-*1*) [1, 3]. The malignant properties of ES have been attributed to EWS/FLI1 proteins acting as aberrant transcription factors [4].

Before chemotherapy became available, only about 10% of patients with ES survived [1]. Advances in multimodality therapy, including aggressive neoadjuvant and adjuvant chemotherapy combined with surgery and/or radiation therapy, have improved long-term survival dramatically, with the 5-year survival of patients with localized disease reaching 70% [3, 5]. Unfortunately, almost 20% of patients have refractory or recurrent disease and approximately one-quarter to one-third present with metastatic disease at diagnosis [1]. Despite many attempts to intensify treatments, survival remains poor in these patients.

Chemotherapy resistance has long been an assiduous challenge for oncologists treating patients with bone sarcomas [6]. Disease recurrence or progression due to treatment resistance of the primary tumor accounted for 60.3% of ES deaths among long-term (≥5-year) survivors in North America who were followed for 20 years posttreatment [7]. However, attempts to attack ES with a higher chemotherapy dose-intensity have produced great morbidity in patients [8]. Therefore, many recent studies have focused on resolving the mechanisms of ES resistance [9–12].

Elucidation of the mechanisms of ES resistance, however, has been impeded by the elusiveness of the cellular origin of ES. Substantial evidence supports a neural cell origin [13–17], while other evidence supports a mesenchymal stem cell origin [18–20]. Analyzing the expression and function of tropomyosin receptor kinase (Trk) family receptors, which are highly expressed in cells of neural origin [21], on ES cells may inform the development of targeted ES therapies.

The endogenous ligands for Trks are neurotrophins, secreted proteins that play a major role in the survival, differentiation, and maintenance of neuronal populations [22]. Neurotrophins also mediate physiological actions outside of the nervous system, including regulating cardiac development, neovascularization, and immune system homeostasis [23]. The four known human neurotrophins — nerve growth factor (NGF), brain-derived neurotrophic factor (BDNF), neurotrophin 3 (NT-3), and neurotrophin 4/5 (NT-4/5) — exert their effects by binding Trk subtypes A, B and C, or binding neurotrophin receptor p75NTR, a member of the tumor necrosis factor receptor superfamily [24]. Trk receptors have been identified as prognostic factors in pediatric malignancies of diverse origins, including neuroblastoma and medulloblastoma [25]. In addition, recent studies have shown that neurotrophins and their receptors are involved in the proliferation, invasiveness, angiogenesis, and drug resistance in various tumor types [25–29].

The potential involvement of neurotrophin receptors in ES has been suggested [21, 30–35], but remains poorly understood. Here, we verified whether Trk receptor inhibition can display anticancer activities in ES cells.

## RESULTS

### Neurotrophin and Trk mRNA expression in cell lines and protein content in tumor samples

**Figure 1:**
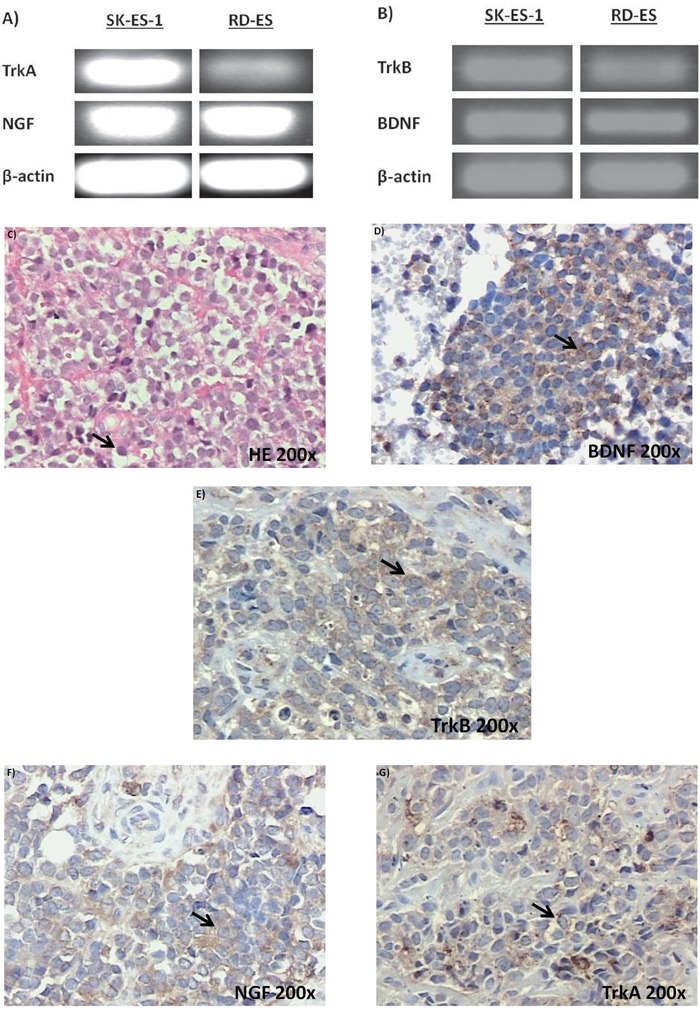
Neurotrophin and Trk mRNA expression in cell lines and protein content in tumor samples **A.** and **B.** Expression of NGF/TrkA and BDNF/TrkB mRNA transcripts in both examined ES cell lines, RD-ES and SK-ES-1. **C.** Representative example of HE-stained section of ES tumor sample demonstrating the small round blue cells that are characteristic of this tumor. **D-G.** Representative photomicrographs of ES sections immunolabeled for BDNF, TrkB, NGF, and TrkA, respectively. Arrows indicate labelled cells.

Reverse transcriptase polymerase chain reaction (RT-PCR) experiments confirmed detectable levels of mRNA transcripts for both NGF and BDNF, as well as for the TrkA and TrkB receptors in SK-ES-1 and RD-ES cell lines (Figure 1A, 1B). Subsequent analyses of immunohistochemically labelled NGF, BDNF, TrkA, and TrkB proteins in a set of seven tumor samples from seven patients with ES revealed heterogenous expression of these proteins across tumor samples (Figure 1C–1G). BDNF was detected in all seven samples, involving, on average, 41.5% of imaged tumor cells. TrkB and NGF proteins were observed on average in 37% and 47% of imaged cells, respectively, in six of the seven samples. TrkA protein was detected in only two samples, in 40% of cells on average. Detailed reporting of the incidence (number of tumors) and distribution (percentage of tumor cells) of labelling for each antigen according to labeling strength/density are reported in detail in Table 1.

**Table 1:** BDNF, TrkB, NGF, and TrkA incidence, distribution, and density in 7 ES tumor samples

Antibody	Expression, N of 7 (%)				
	negative	weak focal	weak difuse	moderate difuse	strong difuse
**BDNF**	0	0	4 (57.1)	3 (42.8)	0
**TrkB**	1 (14.2)	0	5 (71.4)	1 (14.2)	0
**NGF**	1 (14.2)	0	3 (42.8)	3 (42.8)	0
**TrkA**	5 (71.4)	0	1 (14.2)	1 (14.2)	0

### Inhibition of TrkA or TrkB reduce ES cell proliferation

**Figure 2:**
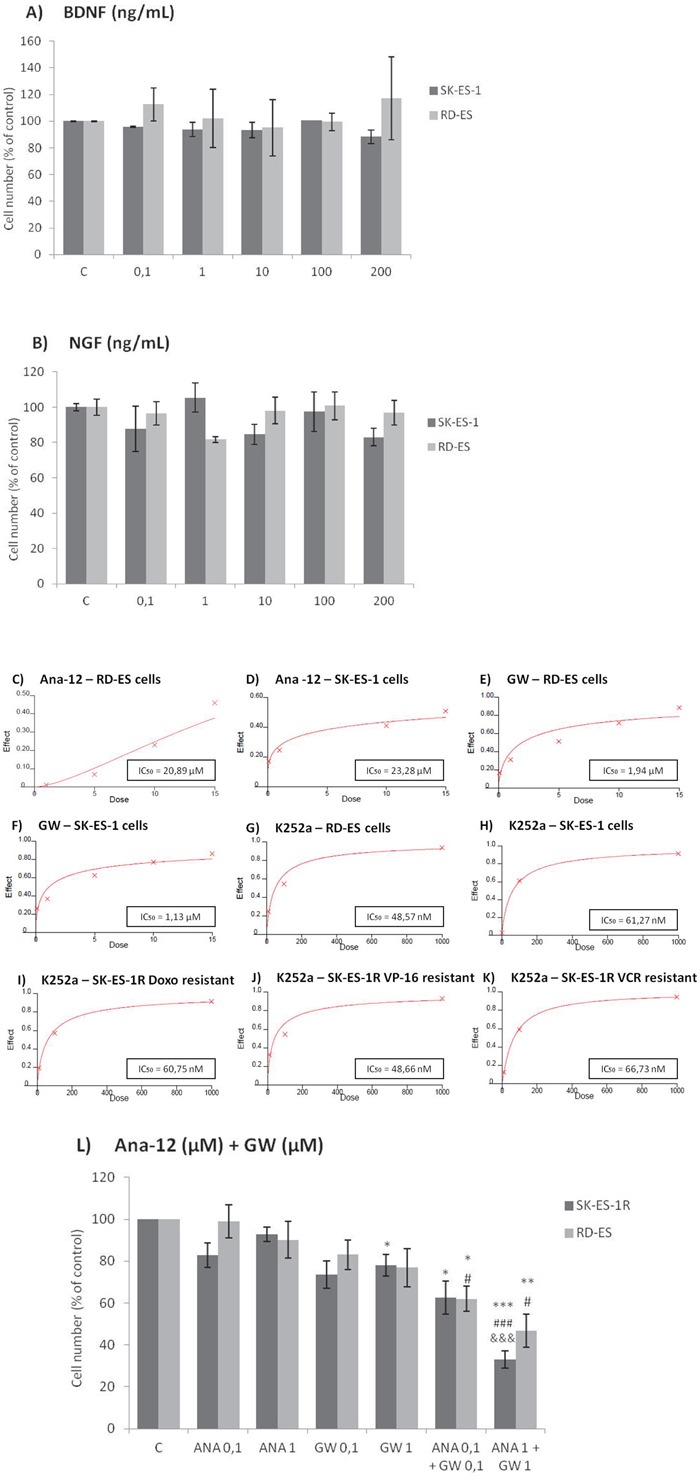
Inhibition of TrkA or TrkB reduces ES cell proliferation **A, B.** Cell proliferation after 72-h treatment with BDNF or NGF (0.1, 1, 10, 100, and 200 ng/mL) in RD-ES and SK-ES-1 cells (n = 3). **C-J.** Dose-response study of the TrkB-specific inhibitor Ana-12 (μM) (C, D) the TrkA-specific inhibitor GW 441756 (μM) (E, F) and the pan-Trk inhibitor K252a (nM) **G-K.** on tumor cell proliferation in human ES RD-ES, SK-ES-1, and SK-ES-1R cell lines. The IC_50_ for each drug was determined by trypan blue counting assay after 72 h treatments. Cell proliferation was assessed in triplicate, in at least three independent experiments. Effect (fraction affected *vs*. control) is plotted on the y-axis versus dose on the x-axis. The linear correlation coefficient *r* of the median-effect plot was >0.90 for all tested agents, ensuring measurement accuracy and conformity to mass-action. Positive controls (100% cell viability) are denoted as ‘0’ effect on the y-axis. **L.** Cell counts following combination treatments of Ana-12 with GW 441756 (0.1 and 1 μM, 72 h; n = 3). * *vs*. control; ^#^ *vs*. respective Ana-12 dose; ^&^ *vs*. respective GW 441756 dose. Single, double, and triple symbols represent *p* < .05, *p* < .01, *p* < .001, respectively.

Cell counting after 72-h treatments of RD-ES and SK-ES-1 cells with a range of doses of BDNF and NGF (0.1, 1, 10, 100, 200 ng/ml) revealed no effects on cell proliferation (Figure 2A, 2B). The lack of effect of BDNF and NGF was also observed under quiescent conditions (data not shown). When SK-ES-1 cells were exposed to the selective BDNF inhibitor Ana-12, there was a significant reduction in cell proliferation, relative to controls, at the doses of 5 μM (*p* < .05), 10 μM (*p* < .01), and 15 μM (*p* < .001; IC_50_ = 23.28 μM) (Figure 2D). Only the 15 μM dose of Ana-12 (*p* < .05) reduced cell proliferation of RD-ES cells significantly (IC_50_ = 20.89 μM) (Figure 2C).

The specific TrkA receptor inhibitor GW 441756 reduced proliferation of SK-ES-1 cells at all doses tested [0.1 μM, (*p* < .01), 1 μM (*p* < .001), 5 μM (*p* < .001), 10 μM (*p* < .001), and 15 μM (*p* < .001; IC_50_ = 1.13 μM)] (Figure 2F) and reduced proliferation of RD-ES cells at all but the lowest dose [1 μM (*p* < 0.05), 5 μM (*p* < 0.01), 10 μM (*p* < .001), and 15 μM (*p* < .001)(IC_50_ = 1.94 μM)] (Figure 2E). It is noteworthy that the IC_50_ values were more than ten times greater for the TrkB receptor inhibitor than for the TrkA receptor inhibitor in both cell lines, indicating higher sensitivity to the TrkA receptor inhibitor.

Inhibition was even more pronounced in both cells with the pan-Trk receptor inhibitor K252a. After 72 h of treatment, SK-ES-1 cell proliferation was decreased, compared to controls, at K252a doses of 100 nM (K100) (*p* < .001) and 1000 nM (K1000) (*p* < .001) (IC_50_ = 61.27 nM) (Figure 2H). In the RD-ES line, reductions in proliferation were also observed with 100 nM (*p* < .001) and 1000 nM (*p* < .001) K252a (IC_50_ = 48.57 nM) (Figure 2G). K252a exhibited an inhibition potency that was almost 20 times higher than that of the TrkA receptor inhibitor GW 441756, which was the more potent selective inhibitor.

When SK-ES-1R cells were exposed to K252a (Figure 2I–2K), the K100 and K1000 groups had reduced cell proliferation, relative to controls, in cells resistant to Doxo (IC_50_ = 60.75 nM), VP-16 (IC_50_ = 48.66 nM), and VCR (IC_50_ = 66.73 nM)(all *p* < .001). The results were similar to those obtained in non-resistant cells, demonstrating that sensitivity to Trk receptor inhibition was retained in the chemoresistant cells.

Combined treatment of Ana-12 and GW 441756 produced more robust inhibition of cell proliferation at 0.1 μM and 1 μM than either inhibitor alone at the same doses in both cell lines (Figure 2L). These results are consistent with the observation of greater effectiveness of the pan-Trk receptor inhibitor K252a compared to selective TrkA and TrkB receptor inhibitors.

### SK-ES-1 cells are affected by specific inhibitors of main pathways activated by Trks

**Figure 3:**
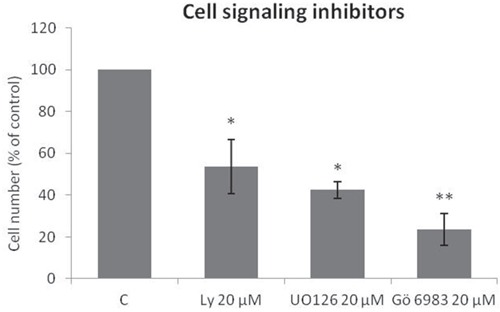
Specific Trk pathway inhibitors reduce SK-ES-1 cell growth Cell proliferation, accessed by cell counting (n = 3), was reduced after 72-h treatment with 20 μM LY294002 (PI3K inhibitor; *p* < .05), UO 126 (MAPK inhibitor *p* < .05), or Gö 6983 (PLCγ/PKC inhibitor; *p* < .01) compared to controls.

The Trk-activated phosphoinositide 3-kinase (PI3K), mitogen-activated protein kinase (MAPK), and phospholipase C-gamma (PLCγ)/protein kinase C (PKC) intracellular signaling pathways are involved in vital cell growth and survival processes [36]. As shown in Figure 3, treatment of ES cells with inhibitors of PI3K (LY294002; *p* < .05), MAPK (UO 126; *p* < .05), or PLCγ/PKC (Gö 6983; *p* < .01) for 72 h resulted in significant reductions in proliferation.

### Cell cycle, morphological, and mRNA expression changes in cells treated with K252a

**Figure 4:**
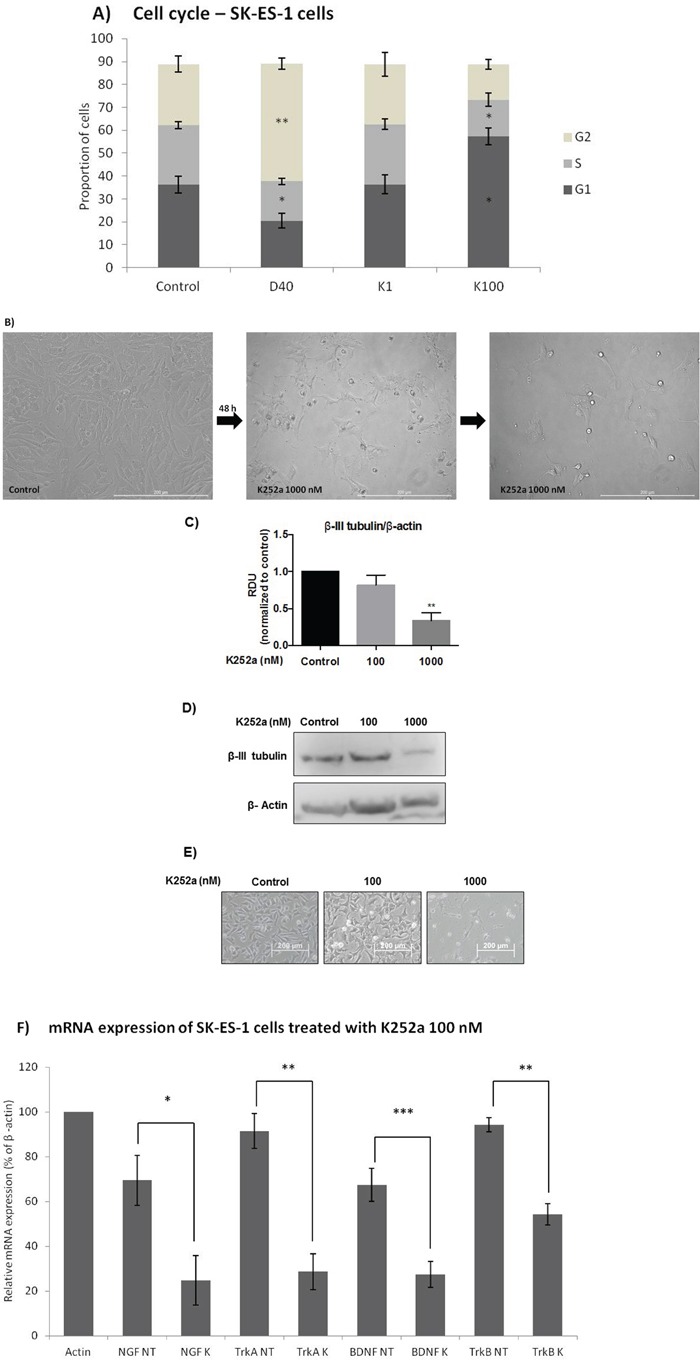
Analyses of cell cycle, morphological changes, neuronal differentiation, and mRNA expression of cells treated with K252a **A.** SK-ES-1 cells were treated with K252a at doses of 1 (K1) and 100 nM (K100) for 24 h. D40 represents 40 nM doxorubicin, positive control. G2-phase and S-phase proportions of cells were increased and decreased, respectively with 100 nM, but not 1 nM, K252a (n =3). **B.** Image displaying morphological differences in SK-ES-1 cells treated with 1000 nM of K252a for 48 h. **C.** Trk inhibition decreases expression of the neural differentiation marker β-III tubulin. Protein levels of β-III tubulin were evaluated by immunoblotting (IB). Relative densitometric analyses were normalized by β-actin and corrected based on vehicle controls. K252a at 100 and 1000 nM induced a mean decrease of 18% and 67% respectively in β-III tubulin relative to controls (*p* < .001; n=3). **D.** Representative Western blot replicate of β-III tubulin levels after 48h of treatment with K252a. β-actin was used for loading control. **E.** Morphology of cells treated with 100 or 1000 nM K252a for 48 h. **F.** The mRNA expression levels of NGF (*p* < .05), TrkA (*p* < .01), BDNF (*p* < .001), and TrkB (*p* < .01) were reduced in SK-ES-1 cells treated with 100 nM K252a for 24 h (K) relative to levels in non-treated (NT) control cells (n = 3).

Flow cytometry cell-cycle analysis after K252a treatment of SK-ES-1 cells for 24 h showed that at 100 nM, but not 1 nM, K252a increased the proportion of G1 cells and decreased the proportion of cells in S phase. Doxo was used as a positive control (Figure 4A). Morphological changes, with possible neurite extensions, were observed in cells exposed to 1000-nM K252a for 48 h (Figure 4B). Moreover, Trk inhibition led to a decrease in the protein expression of β-III tubulin, a neural differentiation marker associated with aggressiveness in tumors. K252a at 100 and 1000 nM induced a mean decrease of 18% and 67% respectively in β-III tubulin relative to controls (Figure 4C–4E).

Significant decreases in the mRNA expression of NGF (*p* < .05), TrkA (*p* < .01), BDNF (*p* < .001), and (*p* < .01) were observed in SK-ES-1 cells treated with 100 nM K252a for 24 h (Figure 4F).

### Antitumor effects of citotoxic chemotherapeutic agents in human ES cell lines

**Figure 5:**
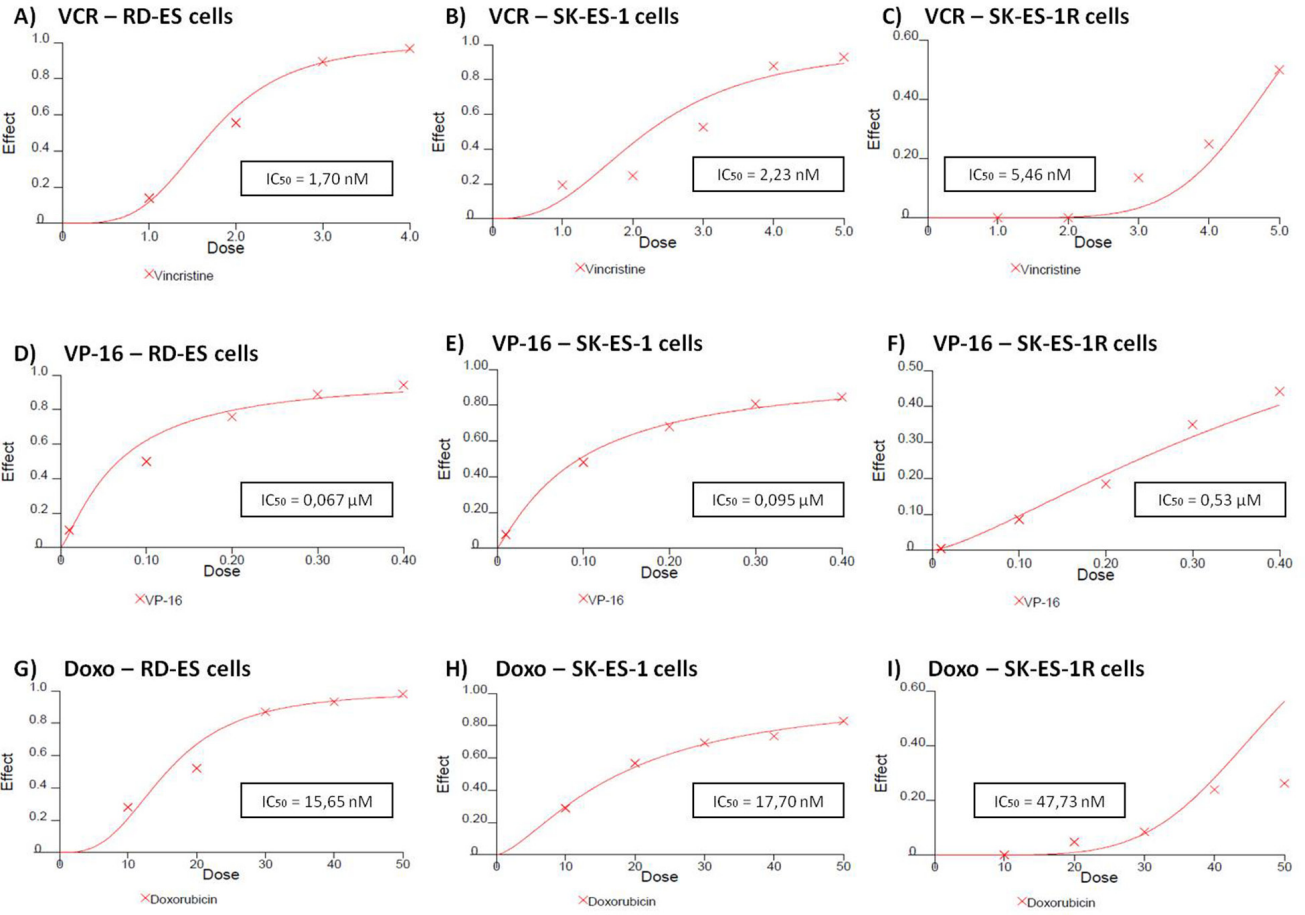
Reduced antitumor effects of VCR (nM) A, B, C. VP-16 (μM) D, E, F. and Doxo (nM) G, H, I. in chemoresistant ES cell line (SK-ES-IR) relative to two non-resistant cell lines Dose-effect IC_50_ concentration curves with drug effect (fraction affected *vs*. control) represented on the y-axis and dose shown on the x-axis. Cell proliferation was assessed with the trypan blue counting assay after 72-h drug treatments in triplicate, in at least three independent series of experiments. Positive controls corresponding to 100% cell viability are denoted as ‘0’ effect on the y-axis. The linear correlation coefficient *r* of the median-effect plot was >0.90 for all tested agents, indicating that the measurements were accurate and had conformity to mass-action. Doxo = doxorubicin; VCR = vincristine; VP-16 = etoposide.

RD-ES, SK-ES-1, and SK-ES-1R cells were exposed to increasing concentrations of standard clinical chemotherapeutic agents, namely vincristine (VCR) (1–5 nM), etoposide (VP-16) (0.1–0.4 μM), and doxorubicin (Doxo) (10–50 nM), for 72 h and trypan blue counting assays were performed (dose-response curves and IC_50_ values are shown in Figure 2). SK-ES-1R cells — in which chemoresistance was induced by the stepwise method (see Materials and Methods) — had significantly higher IC_50_ values (Figure 5C, 5F, and 5I) than non-resistant lines for all three of these chemotherapeutic agents.

### Trk inhibition results in a synergistic enhancement of the antiproliferative effects of chemotherapeutic agents in ES cells

**Figure 6:**
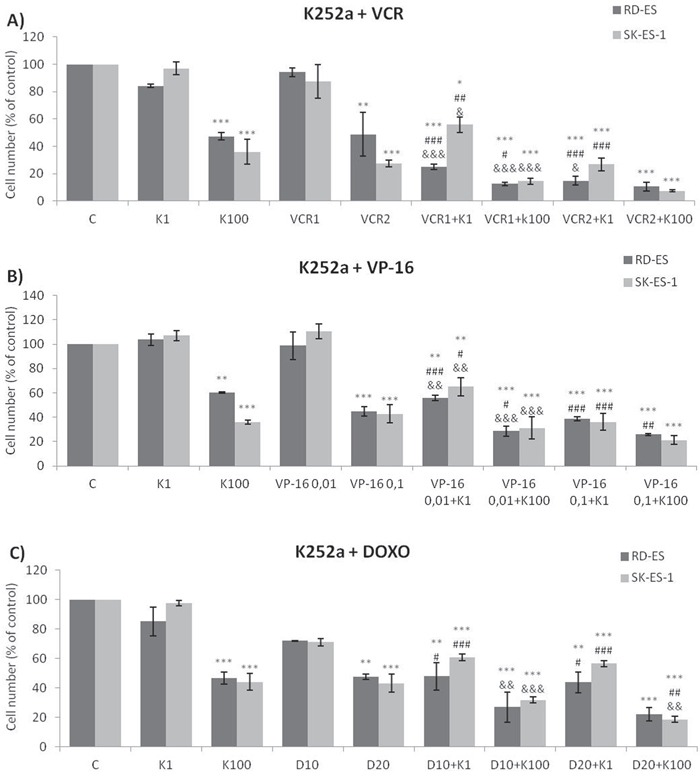
Trk inhibition enhanced the antiproliferative effects of Doxo, VP-16, and VCR synergistically in ES cells **A, B, C.** Proliferation of cells treated with the chemotherapeutic agents VP-16 [0.01 (VP-16 0,01) and 0.1 (VP-16 0,1) μM], VCR [1 (VCR1) and 2 (VCR2) nM], and Doxo [10 (D10) and 20 (D20) nM] alone or in combination with the Trk inhibitor K252a [1 (K1) and 100 (K100) nM]. All combination treatments produced significant decreases compared to controls; some showed further differences versus single treatment. * *vs*. control; ^#^ *vs*. respective K252a dose; ^&^ *vs*. respective chemotherapeutic dose. Single, double, and triple symbols represent *p* < .05, *p* < .01, *p* < .001, respectively. See Table 2 for additional related data. SK-ES-1 treated with VCR (n = 4), VP-16 (n = 3), Doxo (n = 5). RD-ES treated with VCR (n = 3), VP-16 (n = 3), Doxo (n = 4), where n is number of independent experiments contributing to mean data shown. Doxo = doxorubicin; VCR = vincristine; VP-16 = etoposide.

Addition of K252a to a 72-h treatment with a cytotoxic chemotherapeutic agent (VCR, Doxo, or VP-16) resulted in lower cell counts compared to treatments with each chemotherapeutic agent alone (Figure 6). For example, at a 1 nM dose, neither K252a nor VCR affected proliferation significantly. However, when a combined VCR + K252a treatment was used, cell numbers were reduced significantly, with the resultant cell counts being 55% and 25% for SK-ES-1 and RD-ES respectively, of the numbers of cells observed after individual treatments (Table 2).

**Table 2:** Combination index (CI) values calculated from cellular proliferation assays of SK-ES-1 and RD-ES cells treated with K252a, VCR, VP-16, and Doxo alone or in combination

Cell line	Drugs (concentrations)	CI	Interpretation
RD-ES	VCR (1 nM) + K252a (1 nM)	0.440	Synergism
	VCR (2 nM) + K252a (1 nM)	0.608	Synergism
	VCR (1 nM) + K252a (100 nM)	0.734	Moderate synergism
	VCR (2 nM) + K252a (100 nM)	0.891	Slight synergism
	VP-16 (0.01 μM) + K252a (1 nM)	0.188	Strong synergism
	VP-16 (0.1 μM) + K252a (1 nM)	0.531	Moderate synergism
	VP-16 (0.01 μM) + K252a (100 nM)	1.037	Nearly aditive
	VP-16 (0.1 μM) + k252a (100 nM)	1.068	Nearly aditive
	Doxo (10 nM) + K252a (1 nM)	0.629	Synergism
	Doxo (20 nM) + K252a (1 nM)	1.179	Slight antagonism
	Doxo (10 nM) + K252a (100 nM)	0.892	Slight synergism
	Doxo(20 nM) + K252a (100 nM)	1.210	Moderate antagonism
SK-ES-1	VCR (1 nM) + K252a (1 nM)	0.513	Synergism
	VCR (2 nM) + K252a (1 nM)	0.423	Synergism
	VCR (1 nM) + K252a (100 nM)	0.596	Synergism
	VCR (2 nM) + K252a (100 nM)	0.407	Synergism
	VP-16 (0.01 μM) + K252a (1 nM)	0.216	Strong synergism
	VP-16 (0.1 μM) + K252a (1 nM)	0.706	Moderate synergism
	VP-16 (0.01 μM) + K252a (100 nM)	0.644	Synergism
	VP-16 (0.1 μM) + k252a (100 nM)	0.688	Synergism
	Doxo (10 nM) + K252a (1 nM)	0.777	Moderate synergism
	Doxo (20 nM) + K252a (1 nM)	1.364	Moderate antagonism
	Doxo (10 nM) + K252a (100 nM)	1.017	Nearly additive
	Doxo (20 nM) + K252a (100 nM)	0.717	Moderate synergism

### Trk inhibition enhances the antiproliferative effect of chemotherapeutic agents synergistically in ES chemoresistant cells

**Figure 7:**
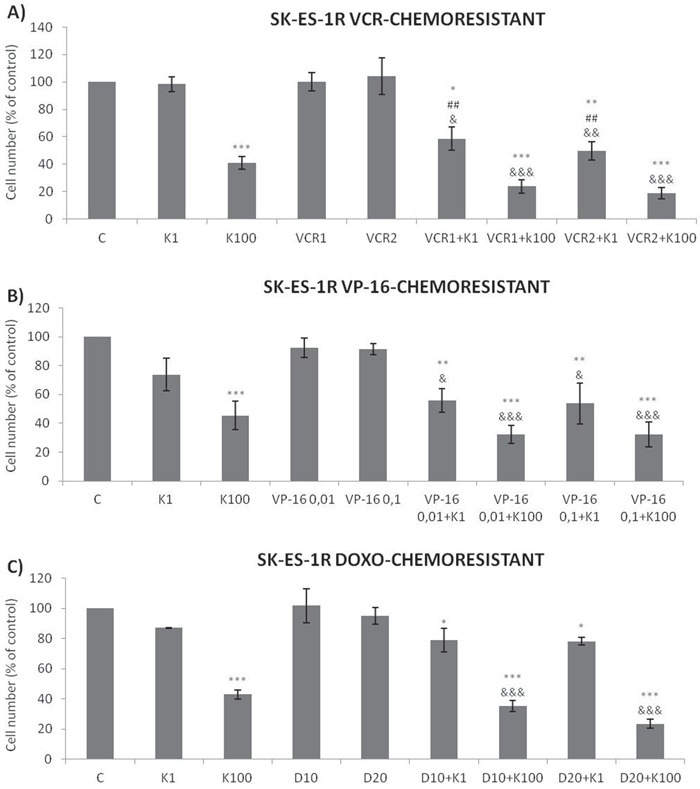
Trk inhibition enhances the antiproliferative effects of VCR, VP-16 and doxorubicin synergistically in chemoresistant ES cells **A-C.** Cells were treated and analyzed as in Figure 6. All adjuvant treatments produced significant reduction in cell proliferation compared to non-treated controls; some differed significant from single treatments. * *vs*. control; ^#^ *vs*. respective K252a dose; ^&^ *vs*. respective chemotherapeutic dose. Single, double, and triple symbols represent *p* < .05, *p* < .01, *p* < .001, respectively. See Table 3 for additional related data. SK-ES-1R treated with VCR (n = 4), VP-16 (n = 3), Doxo (n = 3), where n is number of independent experiments contributing to mean data shown. Doxo = doxorubicin; VCR = vincristine; VP-16 = etoposide.

The antiproliferative effect of K252a in chemoresistant cells was similar to that seen in non-resistant cells (Figure 2). Notably, administration of K252a in combination with each of the tested chemotherapeutic agents (VCR, VP-16, and Doxo) also produced a synergistic antiproliferative effect in chemoresistant cells, indicating that Trk receptor inhibition can re-sensitize cells to chemotherapy (Figure 7, Table 3).

**Table 3:** Combination index values (CI) values calculated from cellular proliferation assays of SK-ES-1R chemoresistant cells treated with K252a, VCR, VP-16, and Doxo alone or in combination

Drugs (concentrations)	CI	Interpretation
VCR (1 nM) + K252a (1 nM)	0.218	Strong synergism
VCR (2 nM) + K252a (1 nM)	0.573	Synergism
VCR (1 nM) + K252a (100 nM)	0.381	Synergism
VCR (2 nM) + K252a (100 nM)	0.588	Synergism
VP-16 (0.01 μM) + K252a (1 nM)	0.094	Very strong synergism
VP-16 (0.1 μM) + K252a (1 nM)	0.697	Synergism
VP-16 (0.01 μM) + K252a (100 nM)	0.229	Strong synergism
VP-16 (0.1 μM) + k252a (100 nM)	0.798	Moderate synergism
Doxo (10 nM) + K252a (1 nM)	0.344	Synergism
Doxo (20 nM) + K252a (1 nM)	0.605	Synergism
Doxo (10 nM) + K252a (100 nM)	0.995	Nearly additive
Doxo (20 nM) + K252a (100 nM)	0.749	Moderate synergism

### Treatment with chemotherapeutics plus K252a co-treatment reduces ES cell colony formation

**Figure 8:**
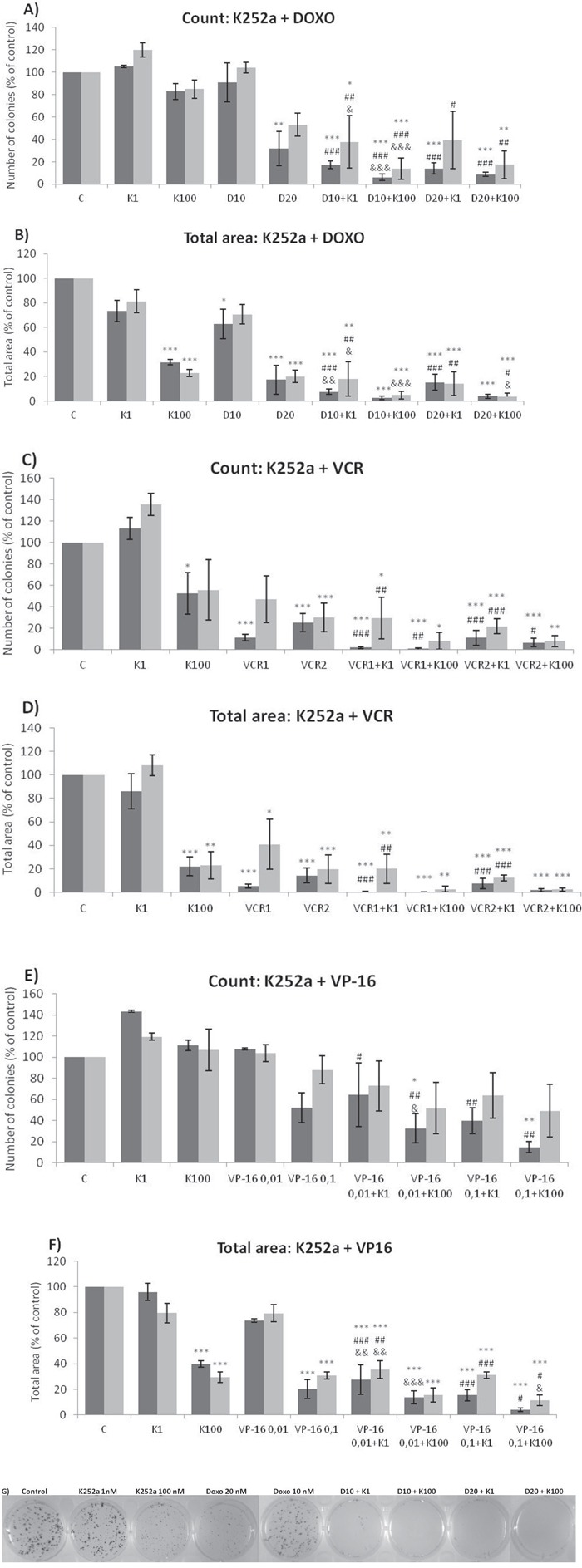
K252a co-treatment with Doxo, VP-16, or VCR reduces ES cell colony formation **A, B.** K252a and Doxo. **C, D.** K252a and VCR. **E, F.** K252a and VP-16. Cells were treated as in Figure 6. Colony count data are shown in (A, C) and (E); colony area data are shown in (B, D) and (F). Mean percentages of three independent experiments are shown. *, **, and *** represent *p* < .05, *p* < .01 and *p* < .001, respectively, *vs*. control; ^#^, ^##^, and ^###^ represent *p* < .05, *p* < .01, and *p* < .001, respectively, *vs*. respective K252a dose; ^&^, ^&&^, and ^&&&^ represent *p* < .05, *p* < .01, and *p* < .001, respectively, *vs*. respective chemotherapeutic dose. **G.** Representative images from cell survival experiments with SK-ES-1 cells treated with Doxo [10 (D10) and 20 (D20) nM] and K252a [1 (K1) and 100 (K100) nM]. Doxo = doxorubicin; VCR = vincristine; VP-16 = etoposide.

Analyses of wells plated with cells that had been treated with drugs and allowed to grow for 10–14 days showed that treatments of K252a combined with either Doxo (Figure 8A, 8B) or VCR (Figure 8C, 8D) reduced the number of colonies formed and the total area occupied by colonies in both SK-ES-1 and RD-ES cell lines. Combined treatment of K252a with VP-16 had a reducing effect on the area occupied by colonies, but not colony number (Figure 8F).

## DISCUSSION

In this report, we showed that the pan-Trk inhibitor K252a can change ES cell morphology, leading to decreased expression of NGF, TrkA, BDNF, and TrkB. K252a reduced the proliferation and survival of ES cells, and produced a synergistic effect when used in combination with chemotherapeutic agents at low doses, even in chemoresistant cells.

Conventional cytotoxic chemotherapy is ineffective in a quarter of patients with localized ES, and in three-quarters of patients with metastatic disease [1]. First-line therapy for localized disease consists of neoadjuvant chemotherapy, which entails a combination of four to six drugs (e.g VCR, Doxo, VP-16, cyclophosphamide, ifosfamide, and dactinomycin) followed by local interventions with surgery and/or radiotherapy when appropriate. Multimodal treatment can improve overall survival (up to 60–70%) in localized disease [37], however this improvement seems to have plateaued. These therapies are being administered at a maximum tolerated intensity. Therefore, raising cure rates may require a more biologically targeted approach, such as one that enhances the effectiveness of current modalities without worsening side effects [38]. Furthermore, relapsed/refractory ES remains uniformly fatal and novel approaches are urgently needed to deal with such cases [39].

Neurotrophins and their receptors play several roles in cancer. Neuroblastoma patients whose tumors have elevated TrkA [40] or TrkC [40, 41] expression have a better prognosis, than those who do not, whereas those with higher TrkB and BDNF levels have a particularly poor prognosis [42]. TrkB expression is also associated with a bad prognosis in patients diagnosed with Wilms tumor [43], but a favorable prognosis in medullary thyroid carcinoma [44].

Some studies have shown that tumor cells treated with BDNF are less sensitive to cytotoxic drugs [26, 45]. Moreover, neurotrophin signaling pathways may function as endogenous systems that protect neurons after biochemical insults, transient ischemia, or physical injury [45, 46]; in other studies, however, BDNF showed anti-cancer potential [47]. Neither BDNF nor NGF alone affected cell proliferation at any of the doses tested here. It is possible that ligand-independent neurotrophin receptor signaling occurs in ES. Alternatively, secretion of endogenous BDNF and NGF by the cells may be enough to activate neurotrophin signaling at optimal levels.

When low doses of selective TrkA and TrkB inhibitors were combined, we observed an increase in antiproliferative effects relative to either inhibitor alone. Also, a similar effect could be reached with nanomolar doses of the pan-Trk receptor inhibitor K252a and was observed in both chemoresistant and non-resistant ES cells. These findings indicate that the combined inhibition of TrkA and TrkB shows higher efficacy compared to inhibiting either receptor alone.

Previous studies in other solid tumor types have indicated that blocking either TrkA or TrkB may produce antitumor effects [29, 36]. For example, Lee and colleagues [48] showed that colorectal tumors positive for TrkA expression presented *NTRK1* rearrengements. In addition, proliferation of *NTRK1*-rearranged patient-derived cells was profoundly inhibited by entrectinib, a pan-TRK inhibitor, and such inhibition was associated with inactivation of TrkA, and down-regulation of downstream signaling pathways.

Low doses of chemotherapeutics with differing mechanisms of action had which had no effect when given alone, but reduced cell proliferation when used together with K252a. A similar effect was seen in a recent study evaluating the efficacy of combining Doxo with an AXL receptor inhibitor (another tyrosine kinase) [49], wherein it was suggested that the synergistic effect depends on the dose and drugs used. Importantly, the fact that similar results were obtained with chemoresistant cells in our study suggests that K252a may be able to subvert general mechanisms of tumor resistance in ES.

Thompson and Levin [50] showed that the morphology of RGC-5 cells (transformed cells expressing surface markers characteristic of neuronal precursor cells similar to ES cells) is changed following treatment with 1000 nM K252a. Similar to the present study, they observed neurite extension following treatment. The fact that this observation occurred in both normal and chemoresistant cells could be critical to understanding the synergy between K252a and chemotherapeutic agents in that differentiated cells can become more sensitive. There may be some specificity to ES cells with respect to cell differentiation given that no morphological changes were observed in fibroblast or feocromacitomas cell lines treated with K252a [50]. However, neuronal differentiation in our study was not confirmed by measuring the content of β-III tubulin, a marker of neuronal differentiation [51]. In fact, a decrease in β-III tubulin levels was observed in cells treated with K252a. Interestingly, increased β-III tubulin has been associated with aggressiveness, resistance to chemotherapy, and poor clinical outcomes in solid tumors [51–54]. β-III tubulin confers dynamic properties to microtubules, likely contributing to resistance to microtubule-targeting chemotherapy [51]. Thus, the decrease in β-III tubulin observed in our study might be related to restoring sensitivity to VCR and K252-induced phenotypic alterations associated with reduced aggressiveness. Further studies are warranted to investigate this interesting possibility.

Activation of Trks leads to stimulation of downstream mediators (i.e. MAPK, PLCγ and PI-3 kinase pathways) important for growth, differentiation, metastasis, and cell survival [36]. BDNF-induced stimulation of TrkB results in increased expression of a wide range of genes, and these alterations are blocked by K252a. In addition, signaling mediated by MAPK is a universal requirement for gene transcription alterations related to Trk activation [55]. Our results indicate that Trk activity regulates the gene expression of Trk receptors as well as their ligands, given that K252a reduced the mRNA levels of NGF, TrkA, BDNF, and TrkB. This transcription inhibition of Trk pathway components represents a likely candidate mechanism involved in the antiproliferative effects of Trk inhibitors.

Only one previous study investigated the immunohistochemical expression of Trk receptors in ES tumors [30]. The authors used a pan-Trk receptor antibody in tumor samples from 5 patients, and found that all samples were positive for at least one Trk receptor. The present study was the first to discriminate between TrkA and TrkB receptor expression, in addition to showing the expression of Trk receptor ligands.

The mechanisms by which ES cells become resistant to chemotherapy are likely multiple and may involve cancer stem cells, proliferative intracellular pathways, and new mutations that allow the tumor cells to escape the effects of chemotherapy [56]. K252a was able to subvert these resistance mechanisms, and produced an excellent long-term response when used in conjunction with chemotherapeutic agents (Figure 8). K252a, an alkaloid-like compound isolated from *Nocardwpsis*, was characterized originally as an inhibitor of PKC and cyclic nucleotide-dependent kinases [57]. It is a potent and selective inhibitor of the tyrosine protein kinase activity of the Trk family of oncogenes and neurotrophin receptors [58]. The pegylated form or K252a (CT 327) has already been tested as a potential treatment for psoriasis in a clinical trial (NCT00995969), and synthetic derivatives of K252a (e.g. CEP-701) have been examined in phase I and phase II studies for leukemia and neuroblastoma [59, 60]. The prior clinical application of these drugs increases the chances that they can be used to treat ES.

Prospective assessment of TrkA and TrkB receptor expression might be used to identify tumors that are likely to respond to Trk receptor inhibitors, either alone or in combination with conventional agents. Indeed, very recent studies have shown that co-administration of Trk receptor inhibitors with traditional chemotherapeutic agents, specific small-interfering RNAs, or radiation enhanced the tumor response greatly in both *in vitro* and *in vivo* models [61–65], supporting the notion that such an approach is a promising avenue for the future of anticancer therapy.

In conclusion, the present results showed that Trk inhibition inhibits ES cell proliferation, particularly when delivered in combination with low-dose chemotherapeutic agents, even in chemoresistant cells. These findings provide the first evidence indicating that Trk pathway inhibition can improve treatment efficacy in ES.

## MATERIALS AND METHODS

### Cell lines and treatments

Human cell lines (SK-ES-1 and RD-ES) were obtained from the American Type Culture Collection (ATCC; Rockville, MD) within six months before the beginning of the experiments and were authenticated using morphology, karyotyping, and PCR based approaches according to standard ATCC procedures. Cells were grown in RPMI-1640 medium (Gibco-BRL, Carlsbad, CA), containing 0.1% Fungizone (250 mg/kg; Invitrogen, São Paulo, Brazil), 100 U/l gentamicin (4 mg/ml; Nova Pharma, Jardim Anápolis, Brazil), 50 mg/ml ampicilin (Nova Pharma, Jardim Anápolis, Brazil), and 10% fetal bovine serum (Invitrogen, São Paulo, Brazil), at 37°C in a humidified incubator under 5% CO_2_. Exponentially growing cells were detached with trypsin 0% EDTA, transferred to culture dishes, and treated accordingly to experimental group designations.

### Resistance induction

To induce chemoresistance, SK-ES-1 cells were cultured with stepwise escalation of concentrations of VCR (0.5, 1.0, 1.5, 2.0, 2.5, 3.0, 3.5, and 4.0 nM), Doxo (1, 5, 10, 15, 20, 25, 30, 35, 40, 45 and 50 nM), and VP-16 (0.01, 0.05, 0.10, 0.20, 0.25, 0.30, 0.35 and 0.4 μM) over 5 months [66]. Cells were exposed to each dose for 15 days. After the highest dose was reached, dose-effect curves were established while the cells were still exposed to the highest dose. The resultant resistant cells were referred to as SK-ES-1R.

### Cellular proliferation assay

Cells were seeded at a density of 2 × 10^4^ cells/well (SK-ES-1) or 2.5 × 10^4^ cells/well (RD-ES and SK-ES-1R) in 24-well plates (TPP, Switzerland). After 24 h, they were treated with K252a (Sigma-Aldrich, St. Louis, MO), VCR, VP-16, Doxo, Ana-12 (Sigma-Aldrich, St. Louis, MO), GW 441756 (Tocris Bioscence, Bristol, UK), BDNF (Sigma-Aldrich, St. Louis, MO) and NGF (Sigma-Aldrich, St. Louis, MO) alone or combined. The medium was removed 72 h after experimental treatments, and the cells were washed with Hanks' balanced salt solution (Invitrogen, São Paulo, Brazil), detached with 0.25% trypsin solution, and counted the trypan blue exclusion method in a hemocytometer [67]. Previous studies from our group [26, 68] have indicated that 72 h is the most appropriate exposure time to assess proliferation in cancer cells treated with K252a. The mean of at least three experiments for each dose, was used to calculate IC_50_ and combination index values.

### Colony formation assay

SK-ES-1 and RD-ES cells were seeded in 6-well plates (500 cells/well) and treated with different doses of K252a, VP-16, Doxo, and VCR, alone or in combination, for 24 h. Subsequently, the drug solution was removed and the cells were placed in a treatment-free medium. After being incubated for 10–14 days, the cells were fixed in 70% ethanol and counterstained with 0.5% crystal violet. Images of each plate were obtained with a desktop scanner (L-pix Chemi Molecular Imaging, Loccus Biotecnologia). Each plate was placed in the same position on the light table by aligning it with the center of the preview exposed light window. For analysis of the clonogenic assay images, optimized digital colony counts were performed with ImageJ software (version 1.37 for Windows) as described by dos Santos et al. [69]. Drug effects were expressed as surviving fraction of the colonies (SF; according to the formula below) and area occupied by colonies. The area occupied by colonies was analyzed in addition to colony number because colony size can vary substantially. All measures were calculated by ImageJ software to ensure uniformity of results.

$$\text{SF}=\frac{\text{No. colonies of treated cells}}{\text{No. untreated control colonies}}\times 100$$

### RT-PCR

SK-ES-1 (chemoresistant and non-resistant cells) and RD-ES cells were cultured in normal RT-PCR medium. Total RNA was extracted with Trizol reagent (Invitrogen, São Paulo, Brazil) in accordance with the manufacturer's instructions and reverse transcribed with superscripttm III First-Strand Synthesis supermix (Invitrogen, São Paulo, Brazil). Human β-actin, BDNF, TrkB, NGF, and TrkA primers were designed according to the corresponding GenBank sequences (Table 4).

Semiquantitative RT-PCR conditions were optimized to determine the number of cycles that would allow product detection within the linear phase of mRNA transcript amplification. Experiments were performed with 1.5 mM MgCl_2_, 0.1 μM for each primer, 0.2 mM DNTPs, 1U Taq Platinum, and 2 μl cDNA template. Expression of β-actin was measured as an internal control. The PCR conditions for β-actin, BDNF, TrkB, NGF, and TrkA experiments were: 2.5 mM MgCl_2_, 0.1 μM for each primer, 0.2 mM DNTPs, 1U Taq Platinum, and 1 μl cDNA template. All assays were carried out in a total volume of 15 μl with 35 amplification cycles that consisted of 1 min at 95°C, denaturation at 94°C for 30 s, annealing at 60°C for 30 s, and primer extension at 72°C for 45 s, followed by a final extension at 72°C for 10 min. The products were electrophoresed in 1.0% agarose gels containing ethidium bromide (Biotium, Hayward, USA) and visualized with ultraviolet light. Fragment lengths were confirmed by reference to a Low DNA Mass Ladder (Invitrogen, São Paulo, Brazil) and relative gene expression was determined by densitometry in ImageJ 1.37 for Windows. Each experiment was performed in triplicate with RNA isolated from independent cell cultures, and representative findings are shown. A negative control was included in each PCR set. Semiquantitative data are shown as percentage changes relative to β-actin (the lowest value among triplicates in the control group was taken as 100%).

**Table 4:** Forward (F) and reverse (R) primers used in RT-PCR amplification

Gene	Primer sequences	Product size (bp)
BDNF	F: 5′ GGCTATGTGGAGTTGGCATT 3′ R: 5′ CTTCAGAGGCCTTCGTTTTG 3′	126
TrkB	F: 5′ TGGTGCATTCCATTCACTGT 3′ R: 5′ CGTGGTACTCCGTGTGATTG 3′	130
NGF	F: 5′ GACTCCGTTCACCCCGTGTGC 3′ R: 5′ CACACCGAGAATTCGCCCCTG 3′	166
TrkA	F: 5′ AACCAGAGCCATGGACTCTACACT 3′ R: 5′ CCCAGCTCTGACAAGCCTCCGA 3′	135
β-actin	F: 5′ GAGACCTTCAACACCCCAG 3′ R: 5′ GCTACAGCTTCACCAGCAG 3′	190

### Flow cytometry cell cycle analysis

SK-ES-1 cells were seeded at the density of 2 × 10^5^ in 6-well plates. The next day, cells were treated with K252a (1 nM or 100 nM) or Doxo (40 nM) as positive control for 24 h. The medium was removed, and the cells were washed with Hanks' balanced salt solution and detached with 0.25% trypsin solution. Cells were centrifuged and re-suspended in 50 μg/mL propidium iodide (Sigma-Aldrich, St. Louis, USA) and 0.1% Triton X-100 in 0.1% sodium citrate solution (appropriate volume to maintain 1 × 10^6^ cells per ml ratio in solution). Cells were incubated on ice for 15 min prior to analysis in an Attune® Acousting Focusing Cytometer (Applied Biosystem, Life Technologies).

### Immunohistochemistry

Paraffin blocks of tumors from 7 patients with ES were obtained from the Hospital de Clínicas de Porto Alegre Pathology Department. Four-micron-thick sections were mounted on organosilane-coated slides and dried overnight at 37°C. The sections were deparaffinized in a stove, rehydrated in graded alcohols, and washed with distilled water. Antigenic recuperation was performed in a microwave, endogenous peroxidases were inactivated by immersion in hydrogen peroxide, and cross-reactivity was blocked with normal serum. Primary antibody [polyclonal rabbit anti-NGF (sc-33603; Santa Cruz Biotechnology), anti-BDNF (sc-20981, Santa Cruz Biotechnology), -TrkB (sc 377218, Santa Cruz Biotechnology), and polyclonal goat anti-TrkA (sc-20539, Santa Cruz Biotechnology)] diluted 1:50 in phosphate-buffered saline was applied for 12 h at 4°C, followed by biotin streptavidin-biotin peroxidase complex (LSAB, Dako). Immunlabelling was visualized by reaction with diamino-9-benzidine tetrahydrochloride (DAB Kit, Dako). Cell nuclei were counterstained lightly with hematoxylin-eosin (HE).

A pathologist (LFRR), who was blind to the group designations scored the immunolabelling semiquantitatively according to intensity and distribution, as described by Scott et al. [70]: for stain intensity, 0 = none; 1 = weak; 2 = moderate, and 4 = strong; and for staining distribution, 1 = focal, <10% of cells and 3 = diffuse, >10% of cells. Tumor sections were considered negative if the sum (intensity + distribution) score was ≤1, weak positive if the sum score was 2–4 (weak diffuse, moderate or strong focal), and strong positive if the sum score was ≥ 5 (moderate or strong diffuse).

### Western blot analysis of β-III tubulin levels

SK-ES-1 ES cells were homogenized in radio-immunoprecipitation assay buffer containing complete Protease Inhibitors (Roche) and quantified using a colorimetric protein assay (Bradford, Bio-rad, CA, USA). 20 μg of total protein lysate were separated by sodium dodecyl sulfate-polyacrylamide gel electrophoresis, transferred to polyvinylidene difluoride membranes, and blotted with antibodies against β-III tubulin (D71G9 – Cell Signaling Technology, MA, USA) and anti-β-actin (A2228, Sigma Aldrich, MO, USA) used as loading control. Incubation with appropriate horsedish peroxidase-conjugated secondary antibody (Santa Cruz, TX, USA) for 1h at RT was performed. Chemiluminescence was detected using ECL Western Blotting substrate (EMD Millipore, DE) and analyzed by ImageQuant LAS500 (GE Healthcare Life Sciences, UK). Densitometric analyses were performed using Image J software (NIH, MD, USA). Relative Densitometric Units (RDU) in controls were expressed as 1 arbitrary unit. Three individual replicates were performed.

### Median dose-effect analysis

The combination index, a measure of synergism and antagonism, was calculated by Chou and Talalay's method with CalcuSyn software version 2.11 for Windows (Biosoft, Ferguson, MO). This method takes into account both potency and dose-effect curve shape. Synergy, additivity, and antagonism were defined as CI < 0.9, CI = 0.9–1.1, and CI > 1.1, respectively. A CI value ≤0.3 and ≤0.1 was interpreted as strong and very strong synergism, respectively [71].

### Statistics

All data are shown as means ± standard errors of the mean (SEM) of 3–5 independent experiments. Differences between mean values were evaluated by one-way analysis of variance (ANOVA) followed by the Tukey-Kramer test in SPSS, version 16.0. P-values < .05 were considered statistically significant.
